# Association of Lower Spiritual Well-Being, Social Support, Self-Esteem, Subjective Well-Being, Optimism and Hope Scores With Mild Cognitive Impairment and Mild Dementia

**DOI:** 10.3389/fpsyg.2018.00371

**Published:** 2018-04-03

**Authors:** Sabrina B. dos Santos, Gabrielli P. Rocha, Liana L. Fernandez, Analuiza C. de Padua, Caroline T. Reppold

**Affiliations:** ^1^Postgraduate Program in Health Sciences, Universidade Federal de Ciências da Saúde de Porto Alegre, Porto Alegre, Brazil; ^2^Department of Psychology, Universidade Federal de Ciências da Saúde de Porto Alegre, Porto Alegre, Brazil; ^3^Department of Basic Health Sciences, Universidade Federal de Ciências da Saúde de Porto Alegre, Porto Alegre, Brazil; ^4^Department of Medical Clinics, Universidade Federal de Ciências da Saúde de Porto Alegre, Porto Alegre, Brazil

**Keywords:** aged, dementia, cognitive decline, well-being, positive psychology, optimism, hope, social support

## Abstract

**Introduction:** Positive psychology (PP) constructs contribute significantly to a better quality of life for people with various diseases. There are still few studies that have evaluated the evolution of these aspects during the progression of dementia.

**Objective:** To compare the scores for self-esteem, life satisfaction, affect, spirituality, hope, optimism and perceived support network between elderly people with mild cognitive impairment (MCI), mild dementia and moderate dementia and control group.

**Methods:** Cross-sectional study. The sample consisted of 66 healthy controls, 15 elderly people with MCI, 25 with mild dementia and 22 with moderate dementia matched by age, gender, and schooling. The instruments used were: Spirituality Self Rating Scale (SSRS), Rosenberg Self-Esteem Scale, Medical Outcomes Study’s Social Support Scale, Life Satisfaction Scale (LSS), Positive and Negative Affect Schedule (PANAS), Revised Life Orientation Test (LOT-R), and Adult Dispositional Hope Scale (ADHS).

**Results:** The scores for spiritual well-being, social support, self-esteem, life satisfaction, positive affect, optimism, negative affect, and hope differed significantly between the groups (*p* < 0.05). The individuals with MCI and mild dementia had lower spiritual well-being, social support, self-esteem, life satisfaction, positive affect, optimism and hope scores, and higher negative affect scores compared with the controls. The scores for PP constructs did not differ between the group of people with moderate dementia and the control group.

**Conclusion:** Dementia was found to impact several PP constructs in the early stages of the disease. For individuals with greater cognitive impairment, anosognosia appears to suppress the disease’s impact on these constructs.

## Introduction

The maintenance of the well-being of elderly people with cognitive impairment is relevant to promote the independence and autonomy of these individuals. One of the demands on health professionals is to prevent or even delay the brain degeneration process, thus contributing to productive aging through the promotion of social engagement and significant interpersonal relationships (Agli et al., 2015; Casemiro et al., 2016). Elderly people exhibit different levels of cognitive impairment. A clinical picture of mild cognitive impairment (MCI) in one or more cognitive domains was recently described in which there was no significant impact on daily living activities. This diagnosis has been incorporated into important classification systems such as APA (American Psychiatric Association, 2014) and National Institute on Aging–Alzheimer’s Association (NIA–AA) (Hyman et al., 2012), which have called it Mild Neurocognitive Disorder and MCI, respectively. This diagnosis is important in that signals a higher likelihood of progression to dementia (Roberts et al., 2014). On the other hand, dementia is a syndrome characterized by acquired cognitive and behavioral symptoms and a decline from previous functioning and performance levels, which interferes with one’s ability to work and engage in usual activities (Hyman et al., 2012). The progressive loss of independence and functionality gives rise to the hypothesis that dementia has a major psychological impact to the subject (Ogawa et al., 2017).

Current studies on emotions have pointed out that individuals with dementia exhibit a lack of recognition, assessment, and even the ability to feel negative emotions (Balconi et al., 2015; Oliver et al., 2015; Bora et al., 2016) and preserve the ability to recognize positive emotions (Goodking et al., 2015; St Jacques et al., 2015). These altered perceptions may be related to the loss of their cognitive ability to understand their disease, known as anosognosia, which impairs their perceptions throughout the course of the dementia, but is associated with an improvement in their quality of life (Conde-Sala et al., 2013, 2014). In order to understand the individual attributes that improve one’s quality of life, Positive psychology (PP) has been consolidated as a field of study interested in promoting well-being and developing individual strengths. PP proposes a change of focus from pathology and the remediation of suffering to positive individual potentials and characteristics (Seligmann and Lopes, 2011).

Positive psychology is the study of the conditions and processes that contribute to the flourishing and optimal functioning of people, groups, and institutions (Gable and Haidt, 2005). The following are the main PP constructs investigated in older people (Snyder and Lopez, 2009): Spirituality: a personal dimension of understanding questions about life, meaning and one’s relationship to the sacred or transcendent (Gonçalves and Pillon, 2009); Social Support: resources made available by a group of people with whom an individual maintains contact and that correspond to certain functions, such as emotional, material, and affective support (Griep et al., 2005); Self-esteem: a visual aspect of self-conception that consists of a set of thoughts and feelings about oneself (Hutz and Zanon, 2011); Optimism: a stable personality trait that gives rise to positive expectations about future events (Scheier et al., 1994); Well-being: a three-part structure in which the first part refers to a cognitive judgment about the degree of satisfaction a person feels about his/her life and the latter two relate to affective components that specify how often a person experiences positive or negative affects (Diener et al., 2004); and Hope: thinking directed to objectives and composed of pathways and agency (Snyder and Lopez, 2009).

Regarding the PP construct approach in dementia, a systematic review that included 11 articles on religion and spirituality in patients with dementia concluded that intervening in these constructs appears to delay cognitive decline and helps them to cope with their disease (Agli et al., 2015). This finding was corroborated by a study by Wu and Koo (2016). These authors conducted a clinical trial with 103 patients with dementia and showed that spiritual interventions improved their hope, life satisfaction, and spiritual well-being. It has been suggested that the social support and sense of belonging that individuals share by being part of the same religious group are the factors that mediate the positive effect of spirituality on patients with dementia (Brewer et al., 2015). Furthermore, Kuiper et al. (2015) reviewed 19 longitudinal cohort studies investigating the association between social relationship and dementia. They concluded that individuals with poor social interaction had a faster decline into dementia. The effect of poor social interaction was similar to that of other well-established risk factors such as limited schooling, physical inactivity, and depression. This result was in line with the work done by Khondoker et al. (2017) in a cohort study that followed 10,055 participants over 10 years to investigate the relationship between dementia and social relationships. The authors concluded that dementia severity showed a significant and negative relationship with the number of bonds the subjects maintained as well as the quality of the social relationships they established. In addition, a systematic review evaluating the effectiveness of social support group interventions for individuals with dementia and MCI suggested that support groups can be psychologically beneficial to people with dementia by lessening their depression and increasing their quality of life (Leung et al., 2015).

Self-esteem in subjects with dementia has also been related to a better quality of life. Higher self-esteem scores predicted a better quality of life in a sample of 95 individuals with dementia and this association was partially mediated by depression and anxiety (Clare et al., 2013). Similar results were observed by Young et al. (2017), who evaluated 57 patient-caregiver dyads in a cross-sectional study and found that self-esteem predicted a higher quality of life for the individuals with dementia.

In addition to self-esteem, other positive variables investigated in patients with dementia are life satisfaction and daily experienced affection. Life satisfaction predicted dementia in a sample of 1.751 elderly people without cognitive impairment at baseline who were followed for 5 years (adjusted OR = 0.70, CI = 0.51–0.96; Peitsch et al., 2016). This dementia risk is also related to negative affects according to a study conducted by Korthauer et al. (2017). The authors assessed affects in 2,137 elderly women without depressive symptoms who were followed for 11 years and concluded that negative affects were associated with greater cognitive decline, even when adjusting for the covariates of age, education, lifestyle, sociodemographic factors, global cognition, cardiovascular risk, and hormone therapy.

The incidence of cognitive impairment was also associated with optimism in a study by Gawronski et al. (2016). These authors evaluated 4,624 elderly people over a period of 4 years and concluded that high optimism was a protective factor for incident cognitive impairment, playing an important role in maintaining cognitive functioning. Likewise, higher levels of hopelessness in midlife were associated with cognitive impairment in a 21-year follow-up with a sample of 200 subjects (Hakanson et al., 2015). Similarly, a systematic review concluded that optimism and hope were beneficial to the treatment of individuals with chronic diseases (Schiavon et al., 2017).

The studies cited above demonstrate that there is great interest in the field of PP in older individuals with cognitive impairment, as they stress the role of many PP constructs as risk factors for this condition and/or the beneficial effects of interventions focusing on these PP constructs on some outcomes, such as quality of life in dementia. Nevertheless, few studies have investigated PP constructs at different levels of cognitive impairment. One investigation described a significant reduction in self-esteem as dementia progressed (Diesfeldt, 2007). Thus, the progression of PP aspects during the course of the disease is still unknown (Wolverson et al., 2009). In view of this, the present study aimed to compare self-esteem, life satisfaction, affect, spirituality, hope, optimism, and perceived support network scores among healthy elderly individuals and subjects with MCI and mild and moderate dementia.

Considering the literature described above, this study’s first hypothesis was that there is a decreasing gradient in all PP constructs among healthy subjects and individuals with MCI and dementia–i.e., better cognitive performance is associated with higher scores for PP constructs. On the other hand, we predicted that individuals with MCI would present lower scores for PP constructs than those with dementia due the latter group’s greater anosognosia–i.e., that the dementia group’s greater cognitive impairment would reduce the individual’s ability to perceive his/her daily limitations, thus affecting the PP constructs.

## Materials and Methods

This is a cross-sectional study. “Cases” were considered those individuals who had some type of cognitive change and “controls” were considered those who were healthy and undergoing a normal aging process. The sample characteristics and inclusion criteria are described below.

### Participants

The sample size was previously calculated in the WinPepi program, version 11.43, based on the studies of Hernandez et al. (2009) and Moreno et al. (2010) resulting in a minimum number of at least 60 demented individuals and 62 healthy controls. The final sample was composed of 128 individuals with a minimum age of 60.

The clinical group (individuals with MCI and dementia) consisted of 62 outpatients who were being treated at a dementia clinic located in a southern Brazilian capital city. Their ages ranged from 60 to 89 (Mean = 72,52; Standard Deviation = 7,92). Dementia diagnoses were made by dementia experts according to the NIA–AA (Hyman et al., 2012), and Brazilian Academy of Neurology criteria (Frota et al., 2011; McKahan et al., 2011). Dementia severity was assessed by the Clinical Dementia Rating (CDR) (Morris, 1993; Chaves et al., 2007). Patients who scored 1 on CDR had mild dementia and who scored 2 presented moderate dementia. Individuals who had a CDR score of 3 (severe dementia) were excluded from the study, as the severity of their impairment would make it impossible for them to understand the questions posed by the instruments that evaluated PP aspects. MCI diagnosis was made by a comprehensive clinical evaluation, which included a clinical history and neuropsychological and functional assessments. Those patients who showed impairment in any cognitive domain and no functional impairment fulfilled the MCI criteria. A CDR score of 0.5 was also used to corroborate a MCI diagnosis. Patients with a current clinical diagnosis of major depression were also excluded. Doctors with clinical experience in evaluating depression and dementia carried out all of the assessments.

The control group consisted of 66 individuals aged 60–88 (Mean = 72,95; Standard Deviation = 7,63) who were selected from a group of elderly people who engaged in physical activities in an active aging group in a capital city in southern Brazil. The controls were chosen after clinical group was selected so that their age, gender, schooling, and social class would be similar to those of the individuals in the clinical group, aiming to match the groups. Depression and cognitive impairment were exclusion criteria for this group. The 15-item Geriatric Depression Scale (GDS) (Sheikh and Yesavage, 1986; Paradela et al., 2005) and the mini mental state examination (MMSE) (Folstein et al., 1975; Bertolucci et al., 1994) were applied to screen for these conditions. Subjects who tested positive for these conditions were not included. All of the subjects were able to perform their day-to-day activities independently.

### Positive Psychology Instruments

The researchers were trained beforehand to apply the PP scales. The following instruments were used to evaluate the PP constructs.

– The Brazilian Portuguese Adaptation of the Spirituality Self Rating Scale (SSRS) (Galanter et al., 2007; Gonçalves and Pillon, 2009). This is a Likert scale composed of six items that assess an individual’s spirituality–i.e., the importance of his/her spiritual dimension and how he/she applies it in his/her life. It is based on three factors: peace, meaning, and faith, and presented adequate internal consistency, with a Cronbach’s alpha of 0.83 (Gonçalves and Pillon, 2009).
– The Brazilian Portuguese Adaptation of the Social Outcomes Study Scale (MOS) (Sherbourne and Stewart, 1991; Griep et al., 2005). This instrument covers five social support dimensions: material, affective, positive social interaction, emotional, and information. It’s a Likert-type scale and presented a Cronbach’s alpha equal to or greater than 0.8 (Griep et al., 2005).
– The Brazilian Version of the Rosenberg Self-Esteem Scale (Rosemberg, 1989; Hutz and Zanon, 2011). This unifactorial instrument consists of ten statements related to a set of feelings of self-esteem and self-acceptance and assesses global self-esteem. It presented a Cronbach’s alpha of 0.90. The items are answered on a 4-point Likert scale, including the following responses: totally agree, agree, disagree, and totally disagree (Hutz and Zanon, 2011).
– The Brazilian Portuguese Version of the Life Satisfaction Scale (LSS) (Diener et al., 1985; Zanon et al., 2014). The LSS scale presented a Cronbach’s alpha of 0.81 and is composed of 5 self-report items arranged on a 7-point Likert scale in which people agree or disagree with the statements to varying degrees (Zanon et al., 2014).
– The Brazilian Version of the Positive and Negative Affect Schedule (PANAS) (Watson and Clark, 1994; Zanon et al., 2013). The Brazilian version of the positive and negative affect schedule presented a Cronbach’s alpha of 0.83 for positive affects and 0.77 for negative affects. It is a self-report scale composed of 10 items that evaluate positive affects and 10 items that evaluate negative affects. The items are adjectives with the answer keys for a Likert five-point scale that correspond to the frequency at which the person experiences the emotion described by the adjective (Zanon et al., 2013).
– The Revised Life Orientation Test (LOT-R) by Scheier et al. (1994) in Its Brazilian Version (Bastianello et al., 2014). Used to evaluate optimism, this scale is composed of a 10-item measure of optimism versus pessimism. Of the 10 items, 3 items measure optimism, 3 items measure pessimism, and 4 items serve as fillers. Respondents rate each item on a 5-point Likert scale with varying degrees of agreement or disagreement. The Cronbach’s alpha of the LOT-R Scale is 0.68 (Bastianello et al., 2014).
– The Brazilian Version of the Adult Dispositional Hope Scale (ADHS) (Snyder et al., 1991; Pacico et al., 2013). The Brazilian version of the adult dispositional hope scale is used to evaluate hope and presented a Cronbach’s alpha of 0.80. This instrument consists of 12 items. Four of these refer to the agency dimension, four refer to the pathway dimension, and four serve as distractors. The items are answered on a 5-point Likert type scale in which 1 is “totally false” and 5 is “totally true” (Pacico et al., 2013).

### Social Class Assessment

Social class was established according to the Economic Classification of the Brazilian Economic Classification Criteria developed by Associação Brasileira de Empresas de Pesquisa [ABEP] (2016), taking into account the population’s purchasing power. This instrument’s final score results in the categories A, B, C, D, and E. Category A includes individuals with an average income of R$ 20,888.00 (Brazilian reals); Category B includes individuals with average incomes between R$ 4,852.00 and R$ 9,254.00; Category C includes individuals with average incomes between R$ 1,625.00 and R$ 2,705.00; and categories D and E include individuals with an average income of R$ 768.00. These amounts are expressed in Brazilian currency, as set forth in the National Household Sample Survey of 2014.

All of the instruments were used in their validated versions for application in Brazil and have good validity and reliability indicators.

### Procedures

Data were initially collected from the individuals in the clinical group. Based on their characteristics (gender, age, schooling, and social class) they were paired with members of the control group when its participants were selected, aiming to match the groups. The elderly people who met the inclusion criteria were invited to participate in the study through an approach carried out in different places, according to the group to be studied. After the informed consent form was explained and signed in a silent and private room, the subjects were evaluated. The PP questionnaires were applied in random order to avoid response bias.

### Ethical Issues

This study was carried out in accordance with the ethical recommendations for clinical research laid out in Resolution 446/12, with written informed consent from all subjects. It was previously approved by the UFCSPA Research Ethics Committee (Protocol No. 1,046,803). All subjects provided informed written consent in accordance with the Helsinki Declaration.

### Statistical Analysis

The data were stored in an SPSS 22.0 database and analyzed using this statistical program. Variable distribution was assessed using the Shapiro–Wilk normality test and variance homogeneity was evaluated with the Levene test. The Student’s *t*-test was used to compare age between the case and control groups; the Chi-square association test was used to compare the other demographic data between the groups. Since the normality and homogeneity tests did not present statistically significant values, parametric tests were applied. To compare the scales between the groups, we applied the one-way analysis of variance (ANOVA) complemented by Tukey’s test. The significance level was set at 5% (*p* < 0.05).

## Results

In total, 128 subjects aged 60–89 (Mean = 72,74; Standard Deviation = 7,50) were included in the study. **Table 1** shows the comparison of the demographic data, presence of cognitive complaints and antidepressant use between the healthy individuals (controls) and those with MCI, mild dementia and moderate dementia (cases). The demographic data (age, schooling, and social class) did not differ between the groups. The MCI and dementia groups presented cognitive complaints. Such complaints became increasingly frequent the higher the level of cognitive impairment and dementia severity. Although major depression was one of the exclusion criteria, some individuals reported that they currently used antidepressants for minor anxiety or depression symptoms. Such use was significantly higher in the control group.

**Table 1:** Comparison of demographic data and complaints of cognitive difficulties between the group of healthy individuals (controls) and the group of people mild cognitive impairment (MCI) and mild/moderate dementia (cases).

		Controls *N* = 66	MCI^+^ *N* = 15	Mild dementia *N* = 25	Moderate dementia *N* = 22	*P*
Age^∗^		72.95 (7.63)	73.27 (8.78)	70.36 (7.39)	74.45 (7.67)	0.316
Gender^∗∗^	*F*	54 (81.8)	11 (73.3)	17 (68.0)	16 (72.7)	0.512
	*M*	12 (18.1)	4 (26.6)	8 (32.0)	6 (27.2)	
Schooling^∗∗^	None	0 (0)	0 (0)	2 (8)	2 (9.1)	0.199
	Primary school education	53 (80.3)	13 (86.7)	16 (64)	17 (77.3)	
	High school education	7 (10.6)	2 (13.3)	5 (20)	3 (13.6)	
	College and/or graduate school	(9.1)	0 (0)	2 (8)	0 (0)	
Social Class^∗∗^	*A*	4 (6.1)	0 (0)	0 (0)	0 (0)	0.522
	*B*	20 (30.3)	3 (20)	5 (20)	5 (22.7)	
	*C*	39 (59.1)	12 (80)	19 (76)	17 (77.3)	
	*D*	3 (4.5)	0 (0)	1 (4)	0 (0)	
Complaint of cognitive difficulty^∗∗^	Yes	24 (36.3)	9 (60.0)	20 (80.0)	20 (90.9)	<0.001
	No	42 (63.6)	6 (40.0)	5 (20.0)	2 (9.0)	
Antidepressant use^∗∗^	Yes	7 (10.6)	3 (20.0)	3 (12.0)	2 (9.0)	0.748
	No	59 (89.3)	12 (80.0)	22 (88.0)	20 (90.9)	

*MCI^+^, Mild Cognitive Impairment. ^∗^ANOVA – results presented as mean and standard deviation. ^∗∗^Chi-square – results presented as n (%)*.

**Table 2** presents a comparison of the scores on the spiritual well-being, social support, self-esteem, life satisfaction, positive affect, negative affect, optimism and hope scales among the healthy elderly people and those with MCI and mild and moderate dementia. There was a significant difference in all of the PP constructs between the groups.

**Table 2:** Comparison of mean and standard deviation in spiritual well-being, social support, self-esteem, life satisfaction, positive affect and negative affect, optimism and hope scores among healthy individuals with mild cognitive impairment and mild/moderate dementia.

	Control Group	Clinical group				
	*N* = 66	Mild cognitive impairment *N* = 15	Mild dementia *N* = 25	Moderate dementia *N* = 22	*P*	*F*_(dfB;dfW)_
Spiritual well-being	39.06 (5.62)^c^	28.00 (6.92)^a^	31.28 (6.42)^a,b^	35.50 (7.89)^b^,c	*<*0.001	17.46_(3;124)_
Peace	12.89 (2.12)^c^	8.6 (2.74)^a^	9.72 (2.33)^a,b^	11.41 (2.55)^b^	*<*0.001	20.70_(3;124)_
Meaning	13.18 (1.92)^c^	8.73 (2.63)^a^	10.60 (2.02)^b^	11.95 (2.69)^b^,c	*<*0.001	21.16_(3;124)_
Faith	13.13 (2.25)^b^	10.67 (3.88)^a^	10.96 (3.90)^a^	12.27 (3.16)^a,b^	0.003	4.85_(3;124)_
Social support	94.74 (11.98)^b^	73.46 (27.02)^a^	74.39 (30.12)^a^	91.18 (18.68)^b^	*<*0.001	9.35_(3;124)_
Material	95.30 (14.43)^b^	82.66 (22.58)^a^	88.20 (17.13)^a,b^	94.09 (15.93)^a,b^	0.029	3.09_(3;124)_
Affective	96.63 (10.92)^b^	80 (27.25)^a^	82.12 (22.67)^a^	92.40 (19.00)^a,b^	*<*0.001	6.43_(3;124)_
Emotional	94.16 (14.89)^b^	74.33 (28.77)^a^	76.8 (26.17)^a,b^	91.59 (19.11)^b^	*<*0.001	7.19_(3;124)_
Information	93.78 (14.54)^b^	68.33 (30.09)^a^	73.2 (28.60)^a^	90.45 (21.03)^b^	*<*0.001	9.86_(3;124)_
Positive social interaction	95.22 (13.34)^b^	63.05 (38.62)^a^	67.43 (33.81)^a^	90.00 (20.23)^b^	*<*0.001	13.51_(3;124)_
Self-esteem	38.10 (2.68)^b^	28.53 (7.92)^a^	30.96 (6.99)^a^	35.95 (4.70)^b^	*<*0.001	23.32_(3;124)_
Life Satisfaction	30.93 (3.73)^b^	25.53 (6.86)^a^	23.24 (5.81)^a^	29.27 (3.79)^b^	<0.001	18.96_(3;124)_
Positive Affect	37.65 (4.88)^b^	28.27 (9.78)^a^	27.00 (7.63)^a^	33.41 (7.95)^b^	<0.001	19.11_(3;124)_
Negative Affect	12.62 (4.60)^a^	25.27 (7.94)^b^	22.24 (7.71)^b^	14.41 (5.47)^a^	<0.001	29.30_(3;124)_
Optimism	24.10 (4.58)^c^	13.87 (6.78)^a^	14.60 (3.96)^a^	20.32 (4.01)^b^	<0.001	36.22_(3;124)_
Hope	36.93 (3.54)^b^	28.40 (7.97)^a^	28.32 (6.01)^a^	34.00 (5.10)^b^	<0.001	24.34_(3;124)_

^∗*a,b,c*^Different letters indicate significant differences (Tukey test at 5% significance); dfB, degrees of freedom between groups; dfW, degrees of freedom within groups.

The multiple comparisons using Tukey’s test showed significantly lower scores for the majority of the PP constructs in individuals with MCI and mild dementia than in the healthy elderly individuals and those with moderate dementia. There was no difference in the total spiritual well-being score between the MCI and mild dementia groups, while individuals with MCI presented significantly lower scores for this construct than the healthy subjects and the individuals with moderate dementia. Multiple comparisons using Tukey’s test also showed that the total social support scores were similar between the MCI and mild dementia groups and that both groups presented lower scores for this construct than the moderate dementia group and the healthy control group. Individuals with MCI showed lower scores in all of this construct’s subgroups than those observed in the healthy group and the moderate dementia group, while the mild dementia group showed lower scores than the moderate dementia group only for the affective, information and positive social interaction sub-scales of social support. Scores for the self-esteem, life satisfaction, positive affect, and hope constructs did not differ between the MCI and mild dementia groups. These constructs were also similar between the moderate dementia group and the healthy individuals. On the other hand, the scores for these constructs were significantly lower in the MCI and mild dementia groups compared with the moderate dementia group and the healthy control group. The healthy controls showed the highest optimism scores among all the groups. The optimism scores did not differ between the mild dementia group and the MCI group and both presented lower levels of optimism than the healthy control group and the moderate dementia group. Negative affect scores were significantly higher in the MCI and mild dementia groups than in the healthy control group, while negative affect levels did not differ between these groups.

## Discussion

The present study evaluated self-perception in several PP constructs in elderly individuals with MCI and different degrees of dementia compared with that in healthy individuals. The study showed two main results: (a) Groups with MCI and mild stages of dementia had worse scores for all constructs, and (b) The scores of patients with moderate-stage dementia did not differ from those of the control group. Therefore, our first hypothesis that better cognitive performance would be associated with higher scores in the PP constructs was refuted. The second hypothesis that cognitive impairment would be associated with better scores in all of the PP constructs was validated.

It is worth discussing some points about the first result (worse scores in the early stages of dementia). It is known that the neuropsychological impacts of dementia include the deterioration of life quality, perception, mood, and behaviors that are associated with neurodegenerative diseases. These changes are mainly perceived at an early stage of the disease and are associated with a poor prognosis of progressive cognitive loss (Ismail et al., 2017). This may explain the first result described. The results of the present study are in partial agreement with those demonstrated by Diesfeldt (2007). This author verified a significant reduction in self-esteem with the progression of dementia. Our results were similar for milder degrees. However, the present study’s assessment found no difference between the controls and the individuals with moderate dementia. This discrepancy may be due to differences in the methods each study used to assess dementia severity.

The reduction of the constructs studied in the earlier stages of dementia can also be understood as resulting from the impacts that the disease generates, posing itself as a threat to existence, the meaning of life and social context, since believing that the rest of one’s life may be unpleasant overwhelms the individual, threatening his/her identity and lowering his/her self-esteem (Cheston et al., 2015). Regarding the wide range of emotions that elderly people with dementia can experience, positive and negative affects are present in varying degrees of intensity in day-to-day life, and it is the relationship between the frequencies with which they are experienced that leads to a greater or lesser feeling of well-being (Diener et al., 2004). In the literature, negative emotions are associated with subsequent dementia. However, since the disease has a long preclinical stage, is difficult to determine whether dementia causes such negative feelings or results from them (Hakanson et al., 2015). What a demented person feels and how he/she feels about the disease is something that has not yet been studied and needs more attention (Schiavon et al., 2017). It is important to point out that even patients with MCI (i.e., whose cognitive impairments do not cause disability) have scores equal to those with mild dementia (worse than the controls’ scores). This could be attributed to their expectation of having an unfavorable progression toward an irreversible and incapacitating disease (Ogawa et al., 2017).

To deal with these disabilities, the literature suggests that dementia interventions in the various positive psychology constructs studied may help the elderly to cope with their disease. Recent studies have investigated the benefits of interventions in positive psychology for individuals with dementia (Coin et al., 2010; Agli et al., 2015; Hakanson et al., 2015; Kuiper et al., 2015; Wu and Koo, 2016; Khondoker et al., 2017). These studies suggest that spirituality in individuals with dementia tends to alleviate or stabilize cognitive disorders (Coin et al., 2010; Agli et al., 2015; Chen et al., 2017) and assists in the development of coping strategies to accept dementia, maintain their relationships, sustain hope, and find meaning in their lives (Jolley et al., 2010; Dalby et al., 2012; MacKinlay, 2012; Agli et al., 2015). Self-esteem interventions are indicated to reduce depressive symptoms and improve life quality (Lee and Park, 2007). Social support and positive relationships can play an important role in maintaining health and preventing dementia (Khondoker et al., 2017), as well as reducing depression (Logsdon et al., 2010; Leung et al., 2015). However, studies are still needed to assess self-esteem, life satisfaction, affect, spirituality, hope, optimism, and support networks in dementia and what effect the disease’s progression has on these aspects.

Our investigation also found that the PP constructs did not differ between the patients with moderate dementia and the healthy controls–i.e., as their dementia severity worsened, the elderly patients’ responses became more positive. This finding is similar to that of a study by Midorikawa et al. (2016) in which family members reported that individuals with mild and moderate dementia displayed more positive behaviors and emotions in their day-to-day lives. Considering the present study’s results, it is hypothesized that this is due to the patient’s perception. Among the constructs evaluated, only optimism continued to show a significant difference between the control group and the group with moderate dementia. A systematic review by Schiavon et al. (2017) emphasized the need for more studies on optimism and hope in people with chronic diseases, highlighting the importance of these constructs in disease prognosis, life satisfaction, and life quality. Regarding emotions, impairment of left-hemisphere emotion regulators (including the left ventrolateral prefrontal cortex, orbitofrontal cortex, and anterior striatal insula) may impair one’s ability to suppress positive emotions such as happiness, thus making individuals more inclined to positive affect (Conde-Sala et al., 2014; Sturm et al., 2015). In advanced stages, there is a functional reduction in these regions as a whole as well as a smaller volume of gray matter in regions of the prefrontal gyrus associated with the recognition of emotions (Stock et al., 2015). This reduction provides a hypothesis for this study’s findings, which indicate that there is no difference in any of the constructs except optimism between the control group and the group with moderate dementia.

As the disease progresses, the perception of its initial impact may not be perceived as a threat, since the disease’s own effects can lead to an inability to judge and understand the losses it causes, as well as to better responses in many domains, such as emotions and empathy (Fong et al., 2016). A patient’s lack of concern about the losses caused by the disease is related to frontal and ventromedial cortical atrophy, which leads him/her to overestimate his/her individual and emotional abilities (Hornberger et al., 2014). Although these losses compromise an individual’s ability to respond to his/her emotions, a qualitative study by Kaufmann and Engel (2016) found that, even with communication challenges, individuals with dementia have the ability to assess their own well-being and affect. The study also pointed to the need for specific methods to evaluate elderly dementia patients to see if they have an adequate understanding of the disease’s impact on their lives. The present study found more frequent complaints of cognitive difficulty in the cases (79%) than in the controls (36.4%). This data suggests that, even if there is a difficulty in understanding the disease, the recognition of cognitive impairment is preserved. Even so, we cannot discard the hypothesis that the greater cognitive impairment of the moderate stage affects judgment and even the comprehension of the existing scales used to evaluate elderly people without dementia. This could also explain the lack of difference in the PP constructs between controls and patients with moderate dementia, in which increased cognitive deficit, anosognosia, and poor understanding of complex issues exert great influence (Poveda et al., 2017).

Greater impairment of an individual’s ability to perceive his/her own limitations with the progression of dementia determines his/her awareness of the pathology’s severity or even of being ill (known as anosognosia), which was mentioned earlier in this study (Conde-Sala et al., 2014; Martyr and Clare, 2017). Studies on anosognosia point to regions of the brain that may be related to this disorder (Perrotin et al., 2015; Arroyo-Anllo et al., 2017). Anosognosia, or the loss of the ability to perceive a disease, makes an individual incapable of responding clearly to his/her emotions or even of perceiving and/or feeling the difficulties experienced in his/her routine. This reduced perception is associated with a serious deficit in the processing and recognition of emotions, which also impairs an individual’s perspective on his/her emotional responses (Poveda et al., 2017). In the literature, anosognosia is associated with the perception of better life quality in advanced stages of dementia (Conde-Sala et al., 2013) and may affect not only cognitive deficits and day-to-day functioning, but also affective symptoms (“affective anosognosia”) described in previous studies (Conde-Sala et al., 2013; Munro et al., 2016). The present study’s findings lead us to believe that anosognosia has a strong influence on an individual’s perception in advanced degrees of dementia, as well as on PP construct scores, highlighting the need for further research in this field in order to better understand the relationships between dementia and emotions.

The scarcity of research on PP and the associations between its constructs and dementia is still evident in current literature (Machado et al., 2017). Considering the results found in the present study, the importance of performing specific evaluations to understand what happens in the various PP constructs during the course of dementia is reinforced.

### Study Strengths

One of the present study’s strengths is that it investigates several PP domains at different stages of dementia, including the earliest stages, such as MCI. It is original in that it provides a view of the differences that exist in PP constructs in terms of the degrees of dementia impairment and performs a broad analysis of the findings, aiming to improve the psychosocial conditions of elderly people.

### Study Limitations

An important limitation of the study is that there is limited literature available in the field of PP, mainly due to the novelty of studies on PP in dementia. Important gaps still exist (Reppold et al., 2015; Freitas et al., 2016). This problem, especially when related to demented individuals and the different stages of dementia, occurs due to the difficulty of evaluating individuals with advanced dementia with existing instruments, since the responses obtained are usually non-specific, non-objective, and strongly influenced by the fluctuations in attention and difficulty in retaining information caused by cognitive impairment (Kaufmann and Engel, 2016).

For future studies, a longitudinal design is suggested with dementia patients in the assessment of the progression of losses in PP, since this may help in understanding the progression of the constructs studied. The impressions of caregivers would be an interesting topic for future studies. Potential causality, selection and measuring biases, and confounding effects are related to the study’s design.

## Conclusion

Mild cognitive impairment and mild dementia result in lower scores for the constructs of self-esteem, spirituality, social support, affect, life satisfaction, optimism, and hope. However, as dementia severity increases, the construct scores more closely resemble those of individuals in the control group, leading to the hypothesis that factors such as anosognosia, a reduction in the suppression of positive emotions and a reduction in emotional self-perception and understanding of the disease gain influence with the progression of dementia.

## Author Contributions

SdS was the lead author in conceptualizing the study and writing the manuscript. GR, AdP, LF, and CR contributed to all stages of the research and then critically reviewed and revised the manuscript. CR was a doctoral advisor for this research. LF and AdP were co-advisors for this research. All authors were accountable for the final version of the manuscript.

## Conflict of Interest Statement

The authors declare that the research was conducted in the absence of any commercial or financial relationships that could be construed as a potential conflict of interest.

## References

[B1] Agli O. Bailly N. Farrand C. (2015). Spirituality and religion in the elderly with dementia: a systematic review. *Int. Psychogeriatr.* 27 715–725. 10.1017/S1041610214001665 25155440

[B2] American Psychiatric Association (2014). *Diagnostic and Statistical Manual of Mental Disorders.* Washington, DC: American Psychiatric Association.

[B3] Arroyo-Anllo E. V. Bouston A. T. Fargeau M. N. Orgaz Baz B. Gil R. (2017). Self-consciousness deficits in Alzheimer’s disease and frontotemporal dementia. *J. Alzheimers Dis.* 55 1437–1443. 10.3233/JAD-160770 27858712

[B4] Associação Brasileira de Empresas de Pesquisa [ABEP] (2016). *Critério Brasil 2015 e Atualização da Distribuição de Classes Para 2016.* Available at: http://www.abep.org/criterio-brasil [accessed January 2 2018].

[B5] Balconi M. Cotelli M. Brambilla M. Manenti R. Cosseddu M. Premi E. (2015). Understanding emotions in frontotemporal dementia: the explicit and implicit emotional cue mismatch. *J. Alzheimers Dis.* 46 211–225. 10.3233/JAD-142826 25757651

[B6] Bastianello M. R. Pacico J. C. Hutz C. S. (2014). Optimism, self-esteem and personality: adaptation and validation of the Brazilian version os the revised life orientation test (LOT-R). *Psico USF* 19 523–531. 10.1590/1413-827120140190030

[B7] Bertolucci P. H. F. Brucki S. M. D. Campacci S. R. (1994). O mini-exame do estado mental em uma população geral: impacto da escolaridade. *Arq. Neuro Psiquiatr.* 52 1–7. 10.1590/S0004-282X1994000100001 8002795

[B8] Bora E. Velakoulis D. Walterfang M. (2016). Meta-analysis of facial emotion recognition in behavioral variant frontotemporal dementia - comparison With Alzheimer disease and healthy controls. *J. Geriatr. Psychiatry Neurol.* 29 205–211. 10.1177/0891988716640375 27056068

[B9] Brewer G. Robinson S. Sumra A. Tatsi E. Gire N. (2015). The influence of religious coping and religious social support on health behaviour, health status and health attitudes in a British Christian sample. *J. Relig. Health* 54 2225–2234. 10.1007/s10943-014-9966-4 25343948

[B10] Casemiro F. G. Rodrigues I. A. Dias J. C. (2016). Impact of cognitive stimulation on depression, anxiety, cognition and functional capacity among adults and elderly participants of an open university for senior citizens. *Rev. Bras. Geriatr. Gerontol.* 19 683–694. 10.1590/1809-98232016019.150214

[B11] Chaves M. L. Camozzato A. L. Godinho C. Kochhann R. Schuch A. de Almeida V. (2007). Validity of the clinical dementia rating scale for the detection and staging of dementia in Brazilian patients. *Alzheimer Dis. Assoc. Disord.* 21 210–217. 10.1097/WAD.0b013e31811ff2b4 17804953

[B12] Chen Y. H. Lin L. C. Chuanq L. L. Chen M. L. (2017). The relationship of physiopsychosocial factors and spiritual well-being in elderly residents: implications for evidence-based practice. *Worldviews Evid. Based Nurs.* 14 484–491. 10.1111/wvn.12243 28510288

[B13] Cheston R. Christopher G. Ismail S. (2015). Dementia as an existential threat: the importance of self-esteem, social connectedness and meaning in life. *Sci. Prog.* 98 416–419. 10.3184/003685015X14467423210693 26790180 PMC10365329

[B14] Clare L. Whitaker C. J. Nelis S. M. Martyr A. Markova I. S. Roth I. (2013). Self-concept in early stage dementia: profile, course, correlates, predictors and implications for quality of life. *Int. J. Geriatr. Psychiatry* 28 494–503. 10.1002/gps.3852 22767455

[B15] Coin A. Perissinotto E. Naijar M. Girardi A. Inelmen E. M. Enzi G. (2010). Does religiosity protect against cognitive and behavioral decline in Alzheimer’s dementia? *Curr. Alzheimer Res.* 7 445–452. 10.2174/156720510791383886 20088813

[B16] Conde-Sala J. L. Reñé-Ramírez R. Turró-Garriga O. Gascón-Bayarri J. Campdelacreu-Fumadó J. Juncadella-Puig M. (2014). Severity of dementia, anosognosia, and depression in relation to the quality of life of patients with Alzheimer disease: discrepancies between patients and caregivers. *Am. J. Geriatr. Psychiatry* 22 138–147. 10.1016/j.jagp.2012.07.001 23567444

[B17] Conde-Sala J. L. Reñé-Ramírez R. Turró-Garriga O. Gascón-Bayarri J. Juncadella-Puig M. Moreno-Cordón L. (2013). Clinical differences in patients with Alzheimer’s disease according to the presence or absence of anosognosia: implications for perceived quality of life. *J. Alzheimers Dis.* 334 1105–1116. 10.3233/JAD-2012-121360 23128559

[B18] Dalby P. Sperlinger D. J. Boddington S. (2012). The lived experience of spirituality and dementia in older people living with mild to moderate dementia. *Dementia* 11 75–94. 10.1177/1471301211416608

[B19] Diener E. Emmons R. Larsen R. Griffin S. (1985). The satisfaction with life scale. *J. Pers. Assess.* 49 91–95. 10.1207/s15327752jpa4901_13 16367493

[B20] Diener E. Scollon C. N. Lucas R. E. (2004). The evolving concept of subjective well-being: the multifaceted nature of happiness. *Adv. Cell Aging Gerontol.* 15 187–219. 10.1016/S1566-3124(03)15007-9

[B21] Diesfeldt H. F. (2007). Measurement of global self-esteem in dementia. Reliability and validity of Brinkman’s self-esteem scale. *Tijdschr. Gerontol. Geriatr.* 38 122–133. 10.1007/BF03074838 17642318

[B22] Folstein M. F. Folstein S. E. McHugh P. R. (1975). “Mini-mental state”: a practical method for grading the cognitive state of patients for the clinician. *J. Psychiatr. Res.* 12 189–198. 10.1016/0022-3956(75)90026-6 1202204

[B23] Fong S. S. Navarrete C. D. Perfecto S. E. Carr A. R. Jimenez E. E. Mendez M. F. (2016). Behavioral and autonomic reactivity to moral dilemmas in frontotemporal dementia versus Alzheimer’s disease. *Soc. Neurosci.* 12 409–418. 10.1080/17470919.2016.1186111 27151065 PMC5651990

[B24] Freitas E. R. Barbosa A. J. G. Neufeld C. B. (2016). Forças do caráter de idosos: uma revisão sistemática de pesquisas empíricas. *Psicol. Pesq.* 10 3–11. 10.24879/201600100020054

[B25] Frota N. A. F. Nitrini R. Damasceno B. P. Forlenza O. Dias-Tosta E. Silva A. B. (2011). Critérios para o diagnóstico de doença de Alzheimer. *Dement. Neuropsychol.* 5 5–10. 10.1590/S1980-57642011DN05030002 PMC5619474 29213739

[B26] Gable S. L. Haidt J. (2005). What (and why) is positive psychology? *Rev. Gen. Psychol.* 9 103–110. 10.1037/1089-2680.9.2.103

[B27] Galanter M. Dermatis H. Bunt G. Williams C. Trujillo M. Steinke P. (2007). Assessment of spirituality and its relevance to addiction treatment. *J. Subst. Abuse Treat.* 33 257–264. 10.1016/j.jsat.2006.06.014 17574800

[B28] Gawronski K. A. B. Kim E. S. Langa K. M. Kubzansky L. D. (2016). Dispositional optimism and incidence of cognitive impairment in older adults. *Psychosom. Med.* 78 819–828. 10.1097/PSY.0000000000000345. 27284699 PMC5349707

[B29] Gonçalves A. M. S. Pillon S. C. (2009). Transcultural adaptation and evaluation of the internal consistency of the Portuguese version of the Spirituality Self Rating Scale (SSRS). *Rev. Psiq. Clín.* 36 10–15. 10.1590/S0101-60832009000100002

[B30] Goodking M. S. Sturm V. E. Ascher E. A. Shdo S. M. Miller B. L. Rankin P. L. (2015). Emotion recognition in frontotemporal dementia and Alzheimer’s disease: a new film-based assessment. *Emotion* 15 416–427. 10.1037/a0039261 26010574 PMC4551420

[B31] Griep R. H. Chor D. Faerstein E. Werneck G. L. Lopes C. S. (2005). Validade de constructo de escala de apoio social do medical outcomes study adaptada para o português no Estudo Pró-Saúde. *Cad. Saúde Publ.* 21 703–714. 10.1590/S0102-311X2005000300004 15868028

[B32] Hakanson K. Soininen H. Winblad B. Kivipelto M. (2015). Feelings of hopelessness in midlife and cognitive health in later life: a prospective population-based cohort study. *PLoS One* 10:e0140261. 10.1371/journal.pone.0140261 26460971 PMC4604196

[B33] Hernandez C. R. Fernándes V. R. Alonso T. O. (2009). Satisfaction with life related to functionality in active elderly people. *Actas Esp. Psiquiatr.* 37 61–67. 19401853

[B34] Hornberger M. Yew B. Gilardoni S. Mioshi E. Gleichqerrcht E. Manes F. (2014). Ventromedial-frontopolar prefrontal cortex atrophy correlates with insight loss in frontotemporal dementia and Alzheimer’s disease. *Hum. Brain Mapp.* 35 616–626. 10.1002/hbm.22200 23125121 PMC6869805

[B35] Hutz C. S. Zanon C. (2011). Revisão da adaptação, validação e normatização da escala de autoestima de Rozemberg. *Aval. Psicol.* 10 41–49.

[B36] Hyman B. T. Phelps C. H. Thomas G. B. Eileen B. H. Nigel J. C. Mario C. C. (2012). National Institute on Aging–Alzheimer’s association guidelines for the neuropathologic assessment of Alzheimer’s disease. *Alzheimers Dement.* 8 1–13. 10.1016/j.jalz.2011.10.007 22265587 PMC3266529

[B37] Ismail Z. Elbaoumi H. Fischer C. E. Hogan D. B. Milliin C. P. Schweizer T. (2017). Prevalence of depression in patients with mild cognitive impairment. A systematic review and meta-analysis. *JAMA Psychiatry* 74 58–67. 10.1001/jamapsychiatry.2016.3162 27893026

[B38] Jolley D. Benbow S. M. Grizzell M. Willmott S. Bawn S. Kingston P. (2010). Spirituality and faith in dementia. *Dementia* 9 311–325. 10.1177/1471301210370645

[B39] Kaufmann E. G. Engel S. A. (2016). Dementia and well-being: a conceptual framework based on Tom Kitwood’s model of needs. *Dementia* 15 774–788. 10.1177/1471301214539690 24948470

[B40] Khondoker M. Rafnsson S. B. Morris S. Orrell M. Steptoe A. (2017). Positive and negative experiences of social support and risk of dementia in later life: an investigation using the English longitudinal study of ageing. *J. Alzheimers Dis.* 58 99–108. 10.3233/JAD-161160 28387667 PMC5438469

[B41] Korthauer L. E. Goveas J. Espeland M. A. Shumaker S. A. Garcia K. R. Tindle H. (2017). Negative affect is associated with higher risk of incident cognitive impairment in nondepressed postmenopausal women. *J. Gerontol.* 10.1093/gerona/glx175 [Epub ahead of print]. 29028908 PMC5861908

[B42] Kuiper J. S. Zuidersma M. Oude Voshaar R. C. Zuidema S. U. van den Heuvel E. R. Stol R. P. (2015). Social relationships and risk of dementia: a systematic review and meta-analysis of longitudinal cohort studies. *Ageing Res. Rev.* 22 3–57. 10.1016/j.arr.2015.04.00 25956016

[B43] Lee Y. M. Park N. H. (2007). The effects of dementia prevention program on cognition, depression, self-esteem and quality of life in the elderly with mild cognitive disorder. *J. Korean Acad. Adult Nurs.* 19 787–796.

[B44] Leung P. Orrel M. Orgeta V. (2015). Social support group interventions in people with dementia and mild cognitive impairment: a systematic review of the literature. *Int. J. Geriatr. Psychiatry* 30 1–9. 10.1002/gps.4166 24990344

[B45] Logsdon R. G. Pike K. C. McCurry S. M. Hunter P. Maher J. Snder L. (2010). Early-stage memory loss support groups: outcomes from a randomized controlled clinical trial. *J. Gerontol. B Psychol. Sci. Soc. Sci.* 65 691–697. 10.1093/geronb/gbq054 20693265 PMC2954328

[B46] Machado F. A. Gurgel L. G. Reppold C. T. (2017). Positive Psychology interventions in adult and elderly rehabilitation: a literature review. *Estud. Psicol.* 34 119–130. 10.1590/1982-02752017000100012

[B47] MacKinlay E. (2012). Resistance, resilience, and change: the person and dementia. *J. Relig. Spirit. Aging* 24 80–92. 10.1080/15528030.2012.633048 28499012

[B48] Martyr A. Clare L. (2017). Awareness of functional ability in people with early-stage dementia. *Int. J. Geriatr. Psychiatry* 32 1–8. 10.1002/gps.4664 28071830

[B49] McKahan G. M. Knopman D. S. Chertkow H. Hman B. T. Jack C. R. Jr. Kawas C. H. (2011). The diagnosis of dementia due to Alzheimer’s disease: recommendations from the national institute on aging-Alzheimer’s association workgroups on diagnostic guidelines for Alzheimer’s disease. *Alzheimers Dement.* 7 263–269. 10.1016/j.jalz.2011.03.005 21514250 PMC3312024

[B50] Midorikawa A. Leyton C. E. Foxe D. Landin-Romero R. Hodges J. R. Piguet O. (2016). All is not lost: positive behaviors in Alzheimer’s disease and behavioral-variant frontotemporal dementia with disease severity. *J. Alzheimers Dis.* 54 549–558. 10.3233/JAD-160440 27472884 PMC5026134

[B51] Moreno J. A. M. Arango-Lasprilla J. C. Rogers H. (2010). Family needs their relationship with psychosocial functioning in caregivers of people with dementia. *Psicol. Caribe.* 26 1–35.

[B52] Morris J. (1993). The clinical dementia rating (CDR): current version and scoring rules. *Neurology* 43 2412–2414. 10.1212/WNL.43.11.2412-a 8232972

[B53] Munro C. E. Donavon N. J. Amariglio R. Papp V. Marshall G. A. Rentz D. M. (2016). The impact of anosognosia and anosodiaphoria on the prediction of progression from mild cognitive impairment to Alzheimer Disease. *Alzheimers Dement.* 12 346–347. 10.1016/j.jalz.2016.06.642

[B54] Ogawa T. Irikawa N. Yanagisawa D. Shiino A. Tooama I. Shimizu T. (2017). Taste detection and recognition thresholds in Japanese patients with Alzheimer-type dementia. *Auris Nasus Larynx* 44 168–173. 10.1016/j.anl.2016.06.010 27427537

[B55] Oliver L. D. Mitchel D. G. V. Dziobek I. MacKinley J. Coleman K. Rankin K. P. (2015). Parsing cognitive and emotional empathy deficits for negative and positive stimuli in frontotemporal dementia. *Neuropsychologia* 67 14–26. 10.1016/j.anl.2016.06.010 25438032

[B56] Pacico J. C. Bastianello M. R. Zanon C. Hutz C. S. (2013). Adaptation and validation of the dispositional hope scale for adolescents. *Psicol. Reflex Crít.* 26 488–492. 10.1590/S0102-79722013000300008 23223324

[B57] Paradela E. M. P. Lourenço R. A. Veras R. P. (2005). Validation of geriatric depression scale in a general outpatient clinic. *Rev. Saúde Pública* 39 918–923. 10.1590/S0034-89102005000600008 16341401

[B58] Peitsch L. Tylas S. L. Menec V. H. St John P. D. (2016). General life satisfaction predicts dementia in community living older adults: a prospective cohort study. *Int. Psychogeriatr.* 28 1101–1109. 10.1017/S1041610215002422 26865088

[B59] Perrotin A. Desgranges B. Landeau B. Mézenge F. La Joie R. Egret S. (2015)). Anosognosia in Alzheimer disease: disconnection between memory and self-related brain networks. *Ann. Neurol.* 78 477–486. 10.1002/ana.24462 26085009

[B60] Poveda B. Osborne-Crowley K. Laidlaw K. Macleod F. Power K. (2017). Social cognition, behaviour and relationship continuity in dementia of the Alzheimer type. *Brain Impair.* 18 175–187. 10.1017/BrImp.2016.35

[B61] Reppold C. T. Gurgel L. G. Schiavon C. C. (2015). Research in positive psychology: a systematic literature review. *Psico USF* 20 275–285. 10.1590/1413-82712015200208

[B62] Roberts R. O. Knopman D. S. Mielke M. M. Cha R. H. Pankratz V. S. Christianson T. J. T. (2014). Higher risk of progression to dementia in mild cognitive impairment cases who revert to normal. *Neurology* 28 317–325. 10.1212/WNL.0000000000000055 24353333 PMC3929198

[B63] Rosemberg M. (1989). *Society and the Adolescent Self-Image*, 2nd Edn Maryand, MD: Princeton University Press.

[B64] Scheier M. F. Carver C. S. Bridges M. W. (1994). Distinguishing optimism from neuroticism (and trait anxiety, self-mastery, and self-esteem): a re-evaluation of the life orientation test. *J. Pers. Soc. Psychol.* 67 1063–1078. 10.1037/0022-3514.67.6.1063 7815302

[B65] Schiavon C. C. Marchetti E. Gurgel L. G. Busnello F. M. Reppold C. T. (2017). Optimism and hope in chronic disease: a systematic review. *Front. Psychol.* 7:2022. 10.3389/fpsyg.2016.02022 28101071 PMC5209342

[B66] Seligmann M. E. P. Lopes C. P. (2011). *Florescer: Uma nova compreensão sobre a natureza da felicidade e do bem-estar. Trad. Cristina Paixão Lopes*, 1st Edn Brasil: Rio de Janeiro.

[B67] Sheikh J. I. Yesavage J. A. (1986). Geriatric depression scale (GDS): recent evidence and development of a shorter version. *Clin. Gerontol.* 5 165–173. 10.1300/J018v05n01_09 20629580

[B68] Sherbourne C. D. Stewart A. L. (1991). The MOS social support survey. *Soc. Sci. Med.* 38 705–714. 10.1016/0277-9536(91)90150-B 2035047

[B69] Snyder C. R. Harris C. Anderson J. R. Holleran S. A. Irving L. M. Sigmon S. T. (1991). The will and the ways: development and validation of an individual-differences measure of hope. *J. Pers. Soc. Psychol.* 60 570–585. 10.1037/0022-3514.60.4.570 2037968

[B70] Snyder C. R. Lopez S. J. (2009). *Psicologia Positiva: uma abordagem científica e prática das qualidades humanas*, 1st Edn Brasil: Artmed. Porto Alegre.

[B71] St Jacques P. L. Grady C. Davidson P. S. Chow T. W. (2015). Emotional evaluation and memory in behavioral variant frontotemporal dementia. *Neurocase* 21 429–437. 10.1080/13554794.2014.917681 24837244

[B72] Stock J. V. De Winter F. L. Gelder B. Ranqarajan J. R. Cypers G. Maes F. (2015). Impaired recognition of body expressions in the behavioral variant of frontotemporal dementia. *Neuropsychologia* 75 496–504. 10.1016/j.neuropsychologia.2015.06.035 26162615

[B73] Sturm V. E. Yokoyama J. S. Eckart J. A. Zakrzewski J. Rosen H. J. Miller B. L. (2015). Damage to left frontal regulatory circuits produces greater positive emotional reactivity in frontotemporal dementia. *Córtex* 64 5–67. 10.1016/j.cortex.2014.10.002 25461707 PMC4346386

[B74] Watson D. Clark L. A. (1994). *The PANAS-X: Manual for the Positive and Negative Affect Schedule - Expanded form.* Available at: https://www2.psychology.uiowa.edu/faculty/clark/panas-x.pdf

[B75] Wolverson E. L. R. Clarke C. Moniz-Cook E. (2009). Remaining hopeful in early-stage dementia: a qualitative study. *Aging Ment. Health* 14 450–460. 10.1080/13607860903483110 20455121

[B76] Wu L. F. Koo M. (2016). Randomized controlled trial of a six-week spiritual reminiscence intervention on hope, life satisfaction, and spiritual well-being in elderly with mild and moderate dementia. *Int. J. Geriatr. Psychiatry* 31 120–127. 10.1002/gps.4300 25965388

[B77] Young D. K. Ng P. Y. Kwok T. (2017). Predictors of the health-related quality of life of Chinese people with major neurocognitive disorders and their caregivers: the roles of self-esteem and caregiver’s burden. *Geriatr. Gerontol. Int.* 17 2319–2328. 10.1111/ggi.13065 28429562

[B78] Zanon C. Bardagi M. P. Layous K. Hutz C. S. (2014). Validation of the satisfaction with life scale to Brazilians: evidences of measurement noninvariance across Brazil and US. *Soc. Indic. Res.* 119 443–453. 10.1007/s11205-013-0478-5

[B79] Zanon C. Bastianello M. R. Pacico J. C. Hutz C. S. (2013). Desenvolvimento e validação de uma escala de afetos positivos e negativos. *Psico USF* 18 193–201. 10.1590/S1413-82712013000200003

